# Dopaminergic signatures of state and trait-like pessimism in mice

**DOI:** 10.64898/2026.07.30.741818

**Published:** 2026-08-05

**Authors:** Srijani Biswas, Steven J. Shabel

## Abstract

The midbrain dopaminergic system is critical for learning reward expectations, but how it contributes to pessimistic bias in reward expectation remains unclear. Using a Pavlovian ambiguous-cue task while recording mesolimbic dopaminergic activity, we find that trial-to-trial, “state” pessimism is associated with decreased dopaminergic activity during ambiguous cues, followed by a self-correcting positive shift in the balance of responses to reward and reward omission. Mice with pessimistically skewed cue interpretation, or “trait-like” pessimism, also had decreased activity during ambiguous cues. However, opposite of state pessimism, trait-like pessimism was associated with a negative shift in outcome signaling driven by larger reward-omission responses. These data indicate that state and trait-like pessimism arise from distinct neural mechanisms and support a model in which trait-like pessimism is maintained by asymmetrically weighted dopaminergic outcome signals.

## Full Text Availability

The license terms selected by the author(s) for this preprint version do not permit archiving in PMC. The full text is available from the preprint server.

